# Self-management Intervention for Reducing Epilepsy Burden Among Adult Ugandans With Epilepsy (Smart-u), Randomised Clinical Trial Protocol

**DOI:** 10.21203/rs.3.rs-3667486/v1

**Published:** 2023-12-02

**Authors:** Scovia Nalugo Mbalinda, Mark Kaddumukasa, Josephine Najjuma, Doreen Birungi, Martin Kaddumukasa, Jennifer Levin, Carolyn Still, Christopher Burant, Avani Modi, Elly. T Katabira, Martha Sajatovic

**Affiliations:** Makerere University; Makerere University; Mbarara University of Science and Technology; Makerere University; Makerere University; University Hospitals Cleveland Medical Center & Case Western Reserve University School of Medicine; Louis Stokes VA Medical Center; Case Western Reserve University; Cincinnati Children’s Hospital Medical Center; Makerere University; University Hospitals Cleveland Medical Center & Case Western Reserve University School of Medicine

**Keywords:** Self-Management, Epilepsy, targeted management, intervention

## Abstract

**Background:**

Epilepsy is a common chronic brain disorder globally affecting people of all ages, with the majority living in developing countries. The introduction of epilepsy self-management approaches to help people with epilepsy is urgently needed to influence epilepsy-related outcomes. This 2-site randomised controlled trial building on promising preliminary data is intended to explore this further.

**Methods:**

A total of 188 adult people with epilepsy (PWE) attending the neurology clinics at Mulago and Mbarara hospitals and consent to participate in the study. They will be randomised into intervention versus enhanced treatment control (eTAU) study groups. The intervention group will receive 12-week “intensive” educational sessions and a 12-week remotely accessed telephone follow-up stage. The controls will continue in their usual care supplemented by written materials on epilepsy in their preferred language and tailored to the reading level of most patients at the clinic. SMART-U consists of 2 main components: a 12-week “intensive” group format stage and a 12-week remotely accessed telephone follow-up stage. SMART-U will be assessed for acceptability, fidelity, and efficacy compared to eTAU. The primary study outcome is the mean change in cumulative past 24-week seizure frequency (24 weeks prior to the study baseline compared to the 24-week follow-up). Seizure frequency will be via self-report with corroboration by family/support system informants whenever possible. Participants will self-report their seizure frequency (numeric count) that they experienced between baseline and 13 weeks and again between 13 and 24 weeks and the mean change from baseline to 24 weeks in QOL.

**Discussion:**

The curriculum-guided Self-Management intervention for Reducing The epilepsy burden among Ugandans (SMART-U) program is anticipated to reduce the epilepsy burden seizure frequency and improve other health outcomes, including depression, functional status and health resource use.

**Trial Registration Number (TRN):**

NCT06139198

**Date of registration:**

14^th^ November 2023

## INTRODUCTION

An estimated 70 million people worldwide carry a diagnosis of epilepsy, and 80% of them live in developing countries[1, 2]. It is estimated that about three-fourths of people with epilepsy in developing countries do not get the treatment they need[1]. Furthermore, people with epilepsy and their families frequently suffer from stigma and discrimination in their communities [1]. In Sub-Saharan Africa (SSA), inadequate care and poor control of seizures often occur, which results in an even greater number of years of potential life loss and a worse burden for patients and families with epilepsy. Poor quality of life impacts patients’ responsibility and often leads to drug non-compliance, exposure to seizure precipitants and poorer disease outcomes. Determining the quality of life among patients with epilepsy will help with developing new strategies for seizure control among these patients. Identifying clinical and demographic factors affecting the quality of life, such as lifestyle changes and early identification and management of these factors, can help improve the quality of life, seizure frequency reduction and better drug compliance.

Epilepsy self-management refers to a patient managing the treatments and day-to-day lifestyle changes associated with epilepsy and greatly influences epilepsy-related outcomes[3, 4]. Provision of care to PWE is multifaceted, including health care workers, nurses and family members. There is a scarcity of neurologists throughout sub-Saharan Africa, especially Uganda, with an estimated one neurologist for every 4 million people in East Africa[5]7. Uganda has about nine active neurologists within urban areas, with none in the rural setting. This makes the provision of care for PWE within both rural and urban settings dire. Utilisation of self-management approaches that may be provided by caregivers, nurses or other family members creates a rare opportunity to provide epilepsy care when healthcare services are difficult to come by and reduce the associated morbidity and mortality rates among this population. Self-management programs like SMART (**S**elf-**ma**nagement fo**r** people wi**t**h epilepsy and a history of negative health events) that were developed to support patients in coping with their chronic condition can improve Qol, reduce perceived stigma, and offer an opportunity to reduce epilepsy-associated mortality and morbidity [6, 7].

While there is a growing body of research to support the use of evidence-based approaches to help people with epilepsy reduce seizures and improve their health [8–10]. There is a scarcity of evidence to prove the effectiveness of self-management programs for people with epilepsy, especially in sub-Saharan Africa, with the majority of the interventions targeting increasing correct knowledge regarding epilepsy [11]. We therefore propose to evaluate the efficacy of the SMART intervention for adults with epilepsy, using a prospective design over a 24-month period in PWE in Uganda.

To advance the care of PWE, it is important to address seizures and QOL, and incorporate available mHealth support for those with poorly controlled epilepsy and limited self-management skills. There are no widely used epilepsy self-management interventions in Uganda. People living in rural communities who are underserved have also been under-studied. Use of widely available mobile phone technology (short message service/SMS) that has been minimally utilised within SSA to assist with seizure surveillance and self-management engagement among PWE.

## Methods

This prospective 2-phase study will use input from relevant stakeholders to refine the SMART-U intervention to ensure acceptability (Phase 1) and then test the effects of the intervention, reducing seizure frequency and improving other health outcomes (Phase 2).

### Study objectives

1. To assess the efficacy of SMART- U vs. eTAU in a prospective randomised controlled trial.

We hypothesised that individuals randomised to SMART-U will have significantly improved QOL with fewer seizures, have greater improvement in depression and functional status compared to eTAU.

1. To use short message service (SMS) delivered via mobile phone text to validate patient self-reported seizure occurrence and push epilepsy self-management messaging in a practical/accessible format.
2. To obtain input from stakeholders (patients, family and clinicians) guided by an Integrated Promotion Action on Research Implementation in Health Services (i-PARIHS) framework to help establish sustainable infrastructure that will facilitate future scale-up of SMART in Uganda with epilepsy partners

### Study setting

The study population will be drawn from neurology clinics where patients with epilepsy and epilepsy-related complications receive free treatment and supportive care at Mulago and Mbarara hospitals. Mulago and Mbarara hospitals are the only hospitals currently that run regular epilepsy clinics that are supported by neurologists in Uganda and tend to provide care for severe forms that are intractable. This offers an opportunity to study this intervention.

At Mulago, patients attending the neurology clinic are referred from medical wards, while others are referred from surrounding health centers for specialised care and treatment for epilepsy. The neurology clinic runs on Wednesdays from 9 am −2 pm with an average attendance of about 5–8 patients with confirmed epilepsy. These patients are then given scheduled visits unless certain illnesses warrant an unscheduled visit. Severely ill patients were referred to the respective wards for admission and in-patient care. A total of about 20–30 patients with epilepsy are seen in the neurology clinic monthly.

Mbarara Regional Referral Hospital is a public regional referral hospital for the districts of Mbarara, Bushenyi, Ntungamo, Kiruhura, Ibanda, and Isingiro, with an official bed capacity of 600. It is located in Mbarara a teaching hospital for the Mbarara University of Science and Technology. MRRH has neurology and psychiatry clinics staffed by physicians and nurses caring for PWE. A neurologist and medical officer run the clinic, equipped with an electroencephalogram with technicians and nurses for diagnosis and care. The clinic receives about 5–10 PWE weekly and 30 PWE monthly.

### Study design

This prospective non-inferiority randomised clinical trial will be conducted at Mulago and Mbarara hospitals. Participants will be adults who meet the inclusion criteria from the epilepsy clinics at Mulago and Mbarara who will be approached to participate in the study and enrolled.

The estimated sample size for the study is 188 people with epilepsy, allowing for 20% attrition or loss by missing data, reducing the final sample to a minimum of N = 150 participants.

Based on a power analysis using G*Power 3.1.9.2121 for a 2 group by 3 time points (baseline, 13-week and 24-week follow-up) repeated measures analysis of variance (RMANOVA) with an alpha of 0.05, a power of. 80, for a sample of 150 we will be able to detect a small standardised effect size of 0.10 for the within-time effect, within the time-by-group interaction effect and a small to moderate standardised effect of 0.19 for the between-group effect.

### Study population

All adult PWE attending the neurology clinics at Mulago and Mbarara hospitals during the study period who or their caregivers will be approached. All participants and their legal guardians will provide written informed consent. All consecutive patients who came either for follow-up or as new referrals with a confirmed diagnosis of epilepsy will be screened for inclusion in the study. We will include those who meet the following inclusion criteria: 1. Participants will have a clinical diagnosis of epilepsy documented with at least two outpatient visits; 2. aged more than 18 years; 3. provide written informed consent by the study participant or immediate caregiver/legal guardian; 4. Ability to participate in study procedures, 5. having had at least 1 seizure in the past 6 months; 6. Owning a mobile phone either by the PWE or immediate caregiver.

We will exclude those 1. Diagnosed with dementia, 2. Participants who are pregnant (given the likely need for different and more intensive treatments among pregnant PWE, may affect their ability to participate in the SMART-U sessions regularly and those unable to participate in study procedures.

### Study procedures

#### Randomisation:

Participants will be randomly assigned in a ratio of 1:1 basis to receive either SMART-U (N = 94) or eTAU (N = 94) using block randomisation. Block randomisation with a random block size of 4 to 8 consecutive patients will be performed to ensure that equal numbers of SMART and ETAU patients occur within strata and are balanced. The randomisation list will be computer-generated by staff from the CWRU Neurological and Behavioural Outcomes Centre Biostatistical Core who are not part of the study staff.

#### SMART-U Intervention group:

The SMART-U self-management approach of the intervention uses nurses and peer educator dyads (PEDs) made up of patients and their care partners to deliver the intervention together. SMART-U begins with a 30–60-minute orientation session where the nurse and PED meet with the patient and their care partner. This is followed by 6 weekly, one-hour group sessions with 6–8 patients and their care partners. Topics covered are noted in Table 1 and are typically delivered over a period of 10–12 weeks. All SMART-U participants continue to receive treatment with their regular medical care providers.

#### Control arm-Enhanced Treatment as Usual (eTAU) group:

Individuals randomised to eTAU will continue in their usual care supplemented by written materials on epilepsy in their preferred language and tailored to the reading level of most patients at the clinic. To control for the same number of patient contacts as SMART-U, the nurse in eTAU will follow up with participants with a series of 8 brief phone calls spaced out over the course of 6 months (approximately every 2 weeks during months 1 and 2, then approximately monthly thereafter).

#### SMS seizure monitoring and support:

During the 24-week duration of the trial, both SMART-U and eTAU participants will receive/use SMS to monitor/report seizure occurrence and duration. Participants will receive an automated weekly survey via SMS asking if they had a seizure in the past week.

#### Feasibility and Fidelity:

Acceptance will be assessed at the end of each series of 8 group sessions with a short self-assessment survey. Following Fraser, fidelity to the SMART-U intervention will be assessed quantitatively and qualitatively. Research staff not involved in the intervention will monitor and score at least 25% of the group sessions, rating each dimension of fidelity on a scale of 1 to 10. The feasibility of texting for both the SMART-U -U and eTAU groups will be assessed by the number of text responses/number of texts sent.

#### Qualitative evaluation:

We will use In-depth interviews on perceived benefits vs. burdens and barriers/facilitators to SMART-U, and eTAU implementation will be conducted at each of the 2 sites. Given the corrosive and persistent nature of stigma on QOL among PWE, input and recommendations on specific strategies regarding ways to potentially mitigate stigma in families and communities will be assessed. Informants will include 20 PWE from SMART-U and 20 PWE from eTAU (total N = 40). We will conduct qualitative interviews to elicit participant perceptions of the intervention at 13-weeks (when SMART-U groups are completed) and again at 24 weeks. For qualitative interviews, this sample size is within the range of recommended sample sizes (i.e., 20–50 individuals).

#### Study outcomes:

The Primary outcome will be Mean change in cumulative past 24-week seizure frequency (24 weeks prior to study baseline compared to the 24-week follow-up) and Mean change from baseline to 24 weeks in QOL. Additional secondary outcomes will include Mental health comorbidity, stigma, epilepsy self-management competency, and other relevant variables, which will be assessed using validated, standardised questionnaires.

#### Study Data:

Study questionnaires will be used for data collection. Study teams will be based within the neurology outpatient clinics; an interviewer will administer the questionnaire and will record answers on the paper questionnaire. Data captured will be entered into a Microsoft Access database by the entry clerk and double-entered to ensure accuracy. Back-up database files will be kept at the end of each data entry session. For quality control purposes, query programs will be written into the database to limit the entry of incorrect data and ensure data entry into the required fields.

#### Data Storage:

Source data in this study will include survey questionnaires and investigation results. All study documents will be kept in secure filing cabinets in the offices at Makerere University, College of Health Sciences. The principal investigator will be responsible for the security of all study documents.

#### Quality Control:

All study team members will be trained in the project objectives, methods of effective communication with study participants and collection of high-quality data. The study team will receive additional training specific to the tasks they will perform within the project, including interview techniques, administration of surveys, and completion of questionnaires. Standard Operating procedures (SOPs) will be written for project activities, and booklets of all relevant documents will be provided to each member of the project team.

#### Data analysis and Data management:

The investigators at each study site will oversee data input and quality and, working with the study statistician and epidemiologist, will oversee the quantitative data analytic procedures. The CWRU data management and statistics team will also provide data management consultation as appropriate. Rigorous development of data collection forms and staff training on properly completing and checking data collection forms will reduce errors at the point of collection. Additional data management practices before and after entry into the RedCap database will identify potential problems or outlying values and catch other errors on data collection forms. Data management staff will be responsible for tracking forms entered and performing routine data checks. Analytic data sets will be prepared using SAS 9.4

Repeated Measure Analysis of Variance. The primary outcomes of interest are quality of life (QOL) and cumulative seizure frequency. Three different analytic strategies will be used to assess AIM 1. Cumulative seizure frequency will be assessed with independent samples t-tests and linear regressions, while QOL and the secondary outcomes will be examined using repeated measures analysis of variance (RMANOVA). We will examine 2 different sets of RMANOVAs.

### Qualitative data analysis plan

Qualitative data analysis. Interview theme analysis will be conducted with accepted qualitative data analysis methods, including specialised software, calculation of inter-rater reliabilities, rater calibration, and systematic synthesis of findings. Additional methodologic detail is provided in the data analysis section.

#### Adverse event identification/classification:

The study investigators and/or qualified research assistants will identify adverse events. All adverse events, whether considered serious or not, will be recorded and reviewed by the study P.I.s on an ongoing basis and reported to the IRB according to local IRB policy. Serious adverse events (A.E.s) are defined as events that result in any of the following: death; a life-threatening experience; in-patient hospitalisation or prolongation of existing hospitalisation; a persistent or significant disability/incapacity; or a congenital anomaly/birth defect (or an event that may require medical or surgical intervention to prevent one of the outcomes listed above).

Seizures, as a defining hallmark of epilepsy, will be recorded if/when they occur but will not be considered either SAEs or A.E.s. All A.E.s, including all SAEs, will be reported to the IRB according to local IRB policy and to the Data Safety and Monitoring Board (DSMB). A summary report of all adverse events will be submitted annually in the report and at the end of the study.

### Data and safety monitoring Board (DSMB) and safety review plan

The study will have a DSMB composed of members who are not part of the study team. At a minimum, they will review and evaluate the accumulated data for participant safety, A.E.s, study conduct and progress every 12 months. Ad-hoc meetings might be called to evaluate unanticipated serious adverse events or any other urgent issues that are relevant and which might occur during the course of the study. The DSMB will be comprised of two clinicians with epilepsy expertise at the Uganda site, a faculty member/clinician with epilepsy expertise at the U.S. site, and a biostatistician at the U.S. site who are all not part of the study team but have extensive experience with federally funded research. The DSMB communication and oversight will be accomplished via videoconference or email communication for issues needing immediate attention.

### Ethics and Dissemination

The study will be conducted according to the Helsinki Declaration[12], the NIH Human Subjects guidelines, and the International Conference on Harmonization E6 Guideline for Good Clinical Practice [13]. Approvals from local leaders will be sought before the study activities commence in the proposed areas. The study nurses will obtain informed consent from potential participants before study procedures are initiated. A copy of the consent form will be given to the study participant. Patient identifiers at the analytic level will differ from the patient’s clinic medical record number. The files that link the patient identifiers to the study numbers will be kept in locked cabinets in the study staff offices. Only aggregate data will be presented, published, and presented such that individual patients cannot be identified. This protocol version 2 dated 29th Sept 2023 was approved by the School of Medicine, Research and Ethics Committee (SOMREC) Mak-SOMREC-2023–648 and UNCST; HS2944ES. The results of this study will be submitted for publication in peer-reviewed journals, and the key findings will be presented at national conferences.

## Discussion

This is the first randomised controlled trial of self-management education for adults with epilepsy in Uganda. The intervention has been adapted for use within the Ugandan health care provision system, and the study aims to provide qualitative and quantitative evidence of the impact of this self-management intervention on the patient’s quality of life and seizure frequency.

The study procedures outlined above are intended to perform a SMART efficacy trial in rural and urban communities while evaluating the barriers and facilitators to self-management in epilepsy.

Innovative and important aspects of the proposed project include the use of a lived experience and provider stakeholder group composed of individuals and integration of mobile services in epilepsy care in Uganda, this study method allows a focus on pragmatic epilepsy outcomes relevant to both PLWE and payers/policymakers including seizure occurrence, stigma, depression, hospitalisations, and self-harm attempts.

## Figures and Tables

**Figure 1:**
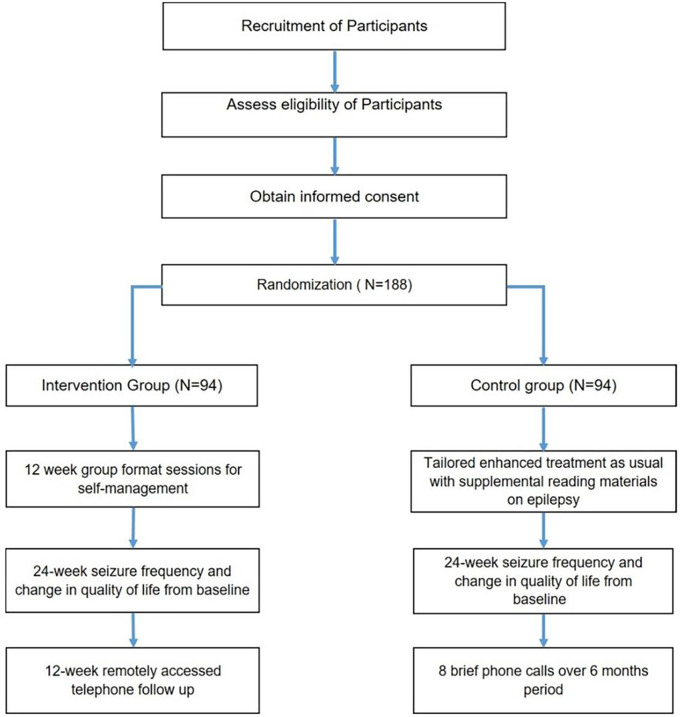
Flow diagram of the study progress through the phases of a parallel randomised trial of two groups (enhanced treatment as usual and SMART-U intervention

**Table 1:** Topics addressed during SMART-U sessions.

Sessions	Activities/Topic
Session 1	Orientation and introductions; Emphasise ground rules; Establishment of a therapeutic relationship; Facts and myths about epilepsy, and general epilepsy management principles
Session 2	Relationship of epilepsy and stress; Stigma and “double stigma”; Strategies to cope with stigma; Introduction to personal goal setting
Session 3	Treatments for epilepsy; Complications of epilepsy; Minimising epilepsy complications; The importance of daily routine and good sleep habits
Session 4	Problem-solving skills and the IDEA approach (Identify the problem, define possible solutions, Evaluate the solutions, Act on the best solution); Talk with your healthcare providers; Role play of communication with care providers.
Session 5	Nutrition for the best physical and emotional health; Substance abuse and its effects on epilepsy; Specific stress–management approaches
Session 6	Effects of exercise and being outdoors on physical and emotional health; Medication routines; Prioritising medication side effects and discussing it with your clinician
Session 7	Social support and using your available supports; Advocacy groups for epilepsy; A personal care plan to take care of the mind and the body
Session 8	Normalising your life in spite of having a chronic but unpredictable condition; Self-management as a lifestyle; Acknowledgment of group process; Setting the stage for ongoing illness management and recovery.

## Data Availability

No datasets were generated or analysed during the current study. All relevant data from this study will be made available upon study completion.

## Abbreviations

DSMB: Data safety monitoring board
eTAU: Enhanced Treatment As Usual
PWE: People with epilepsy
QOL: Quality of life
SMART-U: Self-Management intervention for Reducing The epilepsy burden among Ugandans
SMS: Short messaging service
CWRU: Case Western Reserve University
